# *Azospirillum baldaniorum* Sp245 Induces Physiological Responses to Alleviate the Adverse Effects of Drought Stress in Purple Basil

**DOI:** 10.3390/plants10061141

**Published:** 2021-06-03

**Authors:** Lorenzo Mariotti, Andrea Scartazza, Maurizio Curadi, Piero Picciarelli, Annita Toffanin

**Affiliations:** 1 Department of Agriculture, Food and Environment, University of Pisa, Via del Borghetto 80, I-56124 Pisa, Italy; maurizio.curadi@unipi.it (M.C.); piero.picciarelli@unipi.it (P.P.); annita.toffanin@unipi.it (A.T.); 2 CIRSEC, Centre for Climate Change Impact, University of Pisa, Via del Borghetto 80, I-56124 Pisa, Italy; 3 Interdepartmental Research Center “Nutraceuticals and Food for Health”, University of Pisa, Via del Borghetto 80, I-56124 Pisa, Italy; 4 Institute of Research on Terrestrial Ecosystems (IRET), National Research Council of Italy (CNR), Via Moruzzi 1, I-56124 Pisa, Italy

**Keywords:** *Azospirillum baldaniorum* Sp245, biotic priming, carbon isotope discrimination, drought stress, leaf pigments, *Ocimum basilicum* L., PGPR, photosynthesis, water-use efficiency, phytohormones in xylem sap

## Abstract

*Azospirillum* spp. are plant growth-promoting rhizobacteria (PGPR) that exert beneficial effects on plant growth and yield of agronomically important plant species. The aim of this study was to investigate the effects of a root treatment with *Azospirillum baldaniorum* Sp245 on hormones in xylem sap and physiological performance in purple basil (*Ocimum basilicum* L. cv. Red Rubin) plants grown under well-watered conditions and after removing water. Treatments with *A. baldaniorum* Sp245 included inoculation with viable cells (1·10^7^ CFU mL^–1^) and addition of two doses of filtered culture supernatants (non-diluted 1·10^8^ CFU mL^–1^, and diluted 1:1). Photosynthetic activity, endogenous level of hormones in xylem sap (salicylic acid, jasmonic acid, and abscisic acid), leaf pigments, leaf water potential, water-use efficiency (WUE), and drought tolerance were determined. Fluorescence and gas exchange parameters, as well as leaf water potential, showed that the highest dose of filtered culture supernatant improved both photosynthetic performance and leaf water status during water removal, associated with an increase in total pigments. Moreover, gas exchange analysis and carbon isotope discrimination found this bacterial treatment to be the most effective in inducing an increase of intrinsic and instantaneous WUE during water stress. We hypothesize that the benefits of bacterial treatments based on *A. baldaniorum* Sp245 are strongly correlated with the synthesis of phytohormones and the induction of plant-stress tolerance in purple basil.

## 1. Introduction

*Azospirillum* spp. are notable plant growth-promoting rhizobacteria (PGPR), a group of free-living soil bacteria that exert beneficial effects on plant growth and yield of several crops. The main features of *Azospirillum* spp. are the ability to release phytohormones, fix atmospheric nitrogen, and to enhance root growth, water and mineral uptake, and resistance to drought stress [1]. Production of phytohormones, e.g., abscisic acid (ABA), gibberellins (GAs) and indole-3-acetic acid (IAA), both in culture and in association with the plant, is the mechanism often proposed to explain the effects of *Azospirillum* spp. [2,3]. *Azospirillum* spp. are among the most used PGPR and are commercially available as plant biostimulants, with a growing market in Europe and worldwide [4,5]. *Azospirillum brasilense* Sp245, a strain isolated from external wheat roots that also colonize root xylem as endophyte, has been recently renamed to *Azospirillum baldaniorum* Sp245 [6]. It shows multiple activities, e.g., increasing yield in cereals [7], ABA content in *Arabidopsis* [8], growth in several vegetable crops [9], and drought stress tolerance in wheat and tomato [10]. Although researches mainly focused on cereals (wheat, barley, rice, maize), *Azospirillum* spp. are not cereal-specific at the genus and species levels [9,11] and beneficial effects have been evidenced in the propagation of woody plants, including fruit rootstocks [12] and grapevines [13]. *Azospirillum* spp. also interact with industrial crops (cotton, sunflower), legumes (bean, soybean, chickpea) and vegetable crops (tomato, pepper, cucumber, lettuce) [9], thus making these PGPR potentially valuable for further studies on other plants. 

Sweet basil (*Ocimum basilicum* L.), an annual herbal crop belonging to the Lamiaceae, is an aromatic, medicinal, and culinary plant cultivated worldwide for leaves and essential oils; Italian green-leafed varieties, used for the fresh market and processing, are a typical ingredient of Mediterranean diet [14,15,16]. Purple varieties, such as cv. Red Rubin, accumulate anthocyanins in leaves and are attractive for functional food/pharmaceutical industries [17,18]. Basil is sensitive to drought and a higher anthocyanin content can increase tolerance to water stress [19,20,21,22]. Despite the importance of this crop, studies on basil inoculated with PGPR are lacking. The few reports on basil inoculated with *A. baldaniorum* have mainly studied only productive parameters [23,24] and the effects on resistance to abiotic stress have scarcely been tested [25,26]. The complex environmental challenges facing the agriculture and the increasing global demand for food impose the need to significantly enhance crop productivity within the next few decades [27]. Sustainable agriculture will resort to a greater use of PGPR and transgenic plants in order to increase crop yields in suboptimal conditions, including drought [28,29]. Low water availability is the main environmental factor limiting photosynthesis and crop yields worldwide [30], and priming with PGPR such as *Azospirillum* spp. stimulates drought tolerance in several plants [8,31,32,33].

The aim of our study was to investigate the effects of different treatments with *A. baldaniorum* Sp245 (inoculation with viable cells or addition of two doses of filtered culture supernatants) in plants of purple basil cv. Red Rubin grown in a hydroponic system. The effect of bacterial treatment on drought tolerance was monitored by completely removing water from the system. Comparative analysis of PGPR is challenging because of the diversity of the model systems and considerable variation in the experimental conditions [34]. The hormonal content in xylem sap, i.e., ABA, jasmonic acid (JA), and salicylic acid (SA), leaf pigments content (chlorophylls, total carotenoids and anthocyanins), leaf water potential, gas exchange, and chlorophyll fluorescence were investigated. Moreover, variations in water-use efficiency (WUE) were determined by means of both gas exchanges (intrinsic and instantaneous WUE) and carbon isotope discrimination (Δ) of leaf dry matter and soluble sugars. Although other works have studied the effects of treatment with *A. baldaniorum* on basil physiological performance, to the best of our knowledge this is the first time that hormonal signaling within the xylem sap has been investigated after a root treatment.

## 2. Materials and Methods

### 2.1. Bacterial Strain and Cultivation

Stock cultures of *A. baldaniorum* Sp245 wild-type strain [35] stored at −80 °C in 20% glycerol, were grown in Nutrient agar medium (Oxoid, England). Liquid cultures were grown in NFb medium at 30 °C with gently orbital shaking on an Ika KS 4000 (IKA^®^-Werke GmbH & Co. KG, Staufen im Breisgau, Germany) incubator for approximately 30 h to a CFU value of at least 10^8^ mL^−1^. The NFb media composition was as follows: malic acid 5.0 g L^−1^; K_2_HPO_4_ 0.5 g L^−1^; MgSO_4_⋅7H_2_O 0.2 g L^−1^; NaCl 0.1 g L^−1^; CaCl_2_⋅2H_2_O 0.02 g L^−1^; KOH 4.5 g L^−1^, NH_4_Cl 1 g L^−1^; FeEDTA 4 mL (solution 16.4 g L^−1^); micronutrient solution 2 mL (CuSO_4_⋅5H_2_O 0.04 g L^−1^; ZnSO_4_⋅7H_2_O 0.12 g L^−1^; H_3_BO_3_ 1.40 g L^−1^; Na_2_MoO_4_⋅2H_2_O 1.0 g L^−1^; MnSO_4_⋅H_2_O 1.175 g L^−1^); vitamin solution 1 mL (biotin 100 mg L^−1^; pyridoxal-HCl 200 mg L^−1^); pH to 6.5, as reported by [36], without the addition of bromothymol blue. Cells were harvested by centrifugation (4300× *g* for 10 min, 4 °C), washed in sterile water, and resuspended in a 0.9% NaCl solution. Supernatants were saved and filtered twice by sterile vacuum filtration systems (Merck Millipore, Burlington, MA, USA). The inoculum was set up adjusting the *A. baldaniorum* Sp245 cell number to 10^7^ CFU mL^−1^ with sterile water. The number of viable cells in the inoculum as well as their absence in supernatants was verified by standard plate counts on nutrient agar medium.

### 2.2. Plant Material and Growth Conditions

Seeds of purple basil (*Ocimum basilicum* L.) cv. Red Rubin (Franchi Sementi, Bergamo, Italy) were soaked in distilled water and sown on rock wool (Grodan, Rockwool B.V., The Netherlands) plugs for germination. Each plug, with a single seed, was placed into individual cells in expanded polystyrene trays. For germination, four trays with 60 plugs each were placed on saucers with tap water in a growth chamber under controlled climate conditions (16/8 h light/dark cycles; 400 μmol m^−2^ s^−1^ PPFD; 22 °C). The lamps were high pressure sodium lamps Osram Vialox Nav-T Super 4Y (Osram GmbH, Munich, Germany). Forty basil seedlings were selected for uniform development. Each plug was transplanted into a bigger rock wool block (7.5 cm × 7.5 cm × 6.5 cm, LWH) placed in a plastic pot. Both seedlings and plants were grown according to the non-circulating hydroponic method as described by [37]. Four plastic tanks, each one housing ten transplanted seedlings, were set up for plant growth. Each tank was filled with 5.5 L of the following nutrient solution: Ca(NO_3_)_2_ 0.568 g L^−1^; KNO_3_ = 0.735 g L^−1^; Fe-EDDHA 0.013 g L^−1^; Mg(SO_4_) 0.197 g L^−1^; KH_2_PO_4_ 0.136 g L^−1^; K_2_SO_4_ 0.004 g L^−1^; H_2_SO_4_ 0.125 ml L^−1^; H_3_BO_3_ 0.206 mg L^−1^; CuSO_4_ 0.363 mg L^−1^; ZnSO_4_ 0.871 mg L^−1^; MnSO_4_ 1.552 mg L^−1^; Na_2_MoO_4_ 0.243 mg L^−1^. The nutrient solution level was daily monitored in order to maintain the roots immersed in the solution. Controlled conditions were kept constant throughout the experiment (16/8 h light/dark; 400 μmol m^−2^ s^−1^ PPFD; 22 °C).

### 2.3. Treatments and Inoculation

Four different treatments were prepared and stored in sterile polypropylene flasks until the start of the experiment: C = sterile NFb medium (control); TC = viable cells of *A. baldaniorum* Sp245 1·10^7^ CFU mL^−1^; TS_1_ = filtered culture supernatant from *A. baldaniorum* Sp245 1·10^8^ CFU mL^−1^; TS_2_ = filtered culture supernatant from *A. baldaniorum* Sp245 1·10^8^ CFU mL^−1^, diluted with sterile distilled water (1:1). Each bacterial treatment (0.916 L) was carefully mixed with fresh nutrient solution (4.584 L); the treatment/nutrient solution ratio was 1:5. The total volume in each tank was 5.5 L. The trial consisted of one experiment with ten plants for each bacterial treatment and control (40 plants). Plants were monitored for determination of physiological and biochemical traits at 0 and 7 days after the bacterial treatments (DAT).

### 2.4. Water Stress and Determination of Leaf Water Potential

Seven days after the bacterial treatment (7 DAT) the water was completely removed from the tanks in all the treatments; plants were left without water supply for 6 days, allowing the rock wool block with the root system to dry out gradually. Plants were monitored at 1, 3, and 6 days after stress (DAS). In order to monitor plant water status, leaf water potential (Ψ_w_) was measured before the bacterial treatment (0 DAT), 7 days after the bacterial treatment (7 DAT) and 1, 3, and 6 days after dehydration (1 DAS, 3 DAS, 6 DAS, respectively) by means of a Model 1000 pressure chamber (PMS Instrument Company, Albany, OR, USA) on six leaves per bacterial treatment and control (*n* = 6).

### 2.5. Xylem Sap Sampling and Determination of Hormonal Profiling

Xylem sap was sampled at 0 DAT, 7 DAT, and 6 DAS. Four basil plants per bacterial treatment and control were randomly sampled. Xylem sap samples were collected following a previously-described method [38]. The stem was cut with a fresh razor blade at 10–20 mm above the grodan block and the first droplets were removed with a fluffless cloth to eliminate any phloem exudates [39]. The basal cut end of the stem was tightly inserted into a PVC tube with suitable diameter, and the xylem sap was sampled every 60 min for a total time of 6 h. Xylem sap volumes collected from each plant were measured with a syringe, pooled together in a single sample, and stored at −20 °C until analysis. Xylem sap samples (2 mL) were mixed with [^2^H_4_]-SA (CDN Isotopes Inc., Pointe-Claire, QC, Canada), [^2^H_5_]-JA (CDN Isotopes Inc., Pointe-Claire, QC, Canada) and [^2^H_6_]-ABA (OlChemim Ltd, Olomouc, Czech Republic) as internal standards, acidified (pH = 2.8–3) and thrice partitioned with ethyl acetate (1:1 *v*/*v*). JA, SA, and ABA in the xylem sap were separated by reversed phase HPLC as previously described [40]. Samples were dried, trimethylsilylated with 10 µL of N,O-Bis(trimethylsilyl) trifluoroacetamide (BSTFA) containing 1% trimethylchlorosilane (TMCS) (Pierce, Rockford, IL, USA) at 70 °C for 1 h, and analyzed using GC/MS. Quantitative determination of JA, SA, and ABA was performed using a Saturn 2200 quadrupole ion trap mass spectrometer coupled to a CP-3800 gas chromatograph (Varian Analytical Instruments, Walnut Creek, CA, USA) equipped with a Mega 1MS capillary column (30 m × 0.25 mm i.d., 0.25 µm film thickness) (Mega, Milano, Italy). Plant hormones were identified by comparing full mass spectra with standard compounds. The concentration of each plant hormone in the extracts was calculated by the peak area ratio of labelled and non-labelled ions of internal standard and endogenous hormone, respectively. Final values of hormonal content (nM) in xylem sap were means ± SE (*n* = 3).

### 2.6. Gas Exchange and Chlorophyll Fluorescence Measurements

Gas exchanges were measured using a LI-6400-XT portable photosynthesis system (Li-Cor, Lincoln, NE, USA) on fully expanded and exposed leaves from ten plants for each bacterial treatment and control. Measurements were performed at 0 DAT, 7 DAT, 1 DAS, 3 DAS, and 6 DAS. Instantaneous measurements of steady state photosynthetic CO_2_ assimilation rate (A), stomatal conductance (g_s_), intercellular CO_2_ concentration (C_i_), transpiration rate (E), were performed at light intensity of 400 μmol m^−2^ s^−1^ PPFD, CO_2_ concentration of 400 µmol mol^−^^1^, relative humidity (RH) ranging between 45–55% and leaf temperature of 25 °C. Intrinsic and instantaneous WUE were calculated as A/g_s_ and A/E, respectively. Leaves inside the chamber were allowed to adapt to the above conditions for about 5 min, in order to stabilize gas exchange parameters (steady state values). Chlorophyll a fluorescence was measured on the same plants and leaves used for the gas exchange measurements by means of a miniaturized pulse amplitude-modulated fluorometer (Mini-PAM; Heinz Walz GmbH, Effeltrich, Germany). Leaves were pre-darkened for 30 min before measurements. The potential efficiency of PSII photochemistry (F_v_/F_m_) was calculated on dark-adapted leaves as:

F_v_/F_m_ = (F_m_ − F_0_)/F_m_, (1)

where F_v_ is the variable fluorescence, F_0_ is the minimum fluorescence yield in the dark and F_m_ is the maximum fluorescence yield in the dark after the application of a saturation pulse of light that completely closes all the PSII reaction centres. The actual photon yield of PSII photochemistry (Φ_PSII_) was determined at steady state as follows:

Φ_PSII_ = (F_m_’ − F’)/F_m_’, (2)

where F_m_’ is the maximum fluorescence yield, with all PSII reaction centres in reduced state obtained superimposing a saturating light flash during exposition to actinic light (400 μmol m^−2^ s^−1^) and F’ is the fluorescence at the actual state of PSII reaction centres during actinic illumination. Non-photochemical quenching (NPQ) was determined according to the Stern-Volmer equation [41] as:

NPQ = (F_m_/F_m_’) − 1, (3)

### 2.7. Quantification of Leaf Pigments (Chlorophylls, Total Carotenoids and Total Anthocyanins)

Median leaves (3 per plant) were sampled at 0 DAT, 7 DAT, 3 DAS, and 6 DAS from 3 plants per bacterial treatment and control and stored at −20 °C until processing. Chlorophylls (Chl) and total carotenoids (Car) were extracted as reported in [42]. Leaf tissue (0.5 g of fresh weight, FW) was mixed with anhydrous MgSO_4_ (0.5 g) and sand (0.5 g), added to 4 mL AcOH, ground in mortar and centrifuged (4000× *g* for 10 min). After a 1:15 dilution with AcOH, spectrophotometric analysis was performed at 661.6 nm for chlorophyll a (Chl a), 644.8 nm for chlorophyll b (Chl b) and 470 nm (Car) using a Shimadzu UV-1800 spectrophotometer (Shimadzu Corporation, Kyoto, Japan). Chl (a, b, total) and Car were quantified according to [43]. Total anthocyanins (Ant) were extracted as described in [15]. Leaves (1 g FW) were added (1:10 *w*/*v*) with MeOH acidified with HCl 0.2 M (85:15 *v*/*v*); the solution was adjusted to pH 1 with 4N HCl, ground in a mortar, and centrifuged (4000× *g* for 10 min). After a 1:10 dilution with acidified MeOH, the absorbance was measured at 520 nm. Total anthocyanins were expressed as cyanidin 3-O-glucoside chloride equivalents [44]. Final values of leaf pigments (mg g^−1^ of dry weight, DW) were means ± SE (*n* = 3).

### 2.8. Carbon Isotope Discrimination of Leaf Dry Matter and Soluble Sugars

Three fully-expanded and exposed leaves were sampled from 5 plants of each bacterial treatment and control at 6 DAS and used for the determination of carbon isotope discrimination (Δ) of leaf dry matter (Δ_dm_) and total leaf soluble sugars (Δ_ss_). Leaves were immediately frozen, freeze-dried and finely ground in mortar. An aliquot (100 mg) of the powder was used for soluble sugars extraction, according to [45]. Samples were suspended in 5 mL of demineralized water and shaken for 40 min at room temperature. After centrifugation at 12,000× *g*, supernatants were purified with Dowex-50 (H^+^) resin for separation of amino acids from organic acids and sugars, and with Dowex-1 (Cl^−^) resin for separation of organic acids from sugars. Sugar-containing fractions were freeze-dried and stored before carbon isotope analysis. About 1–2 mg of soluble sugars and leaf dry matter were quantitatively combusted into an elemental analyzer (Model NA 1500, Carlo Erba, Milan, Italy) and the carbon dioxide produced was admitted in helium stream to an isotope ratio mass spectrometer (Isoprime Ltd., Cheadle, UK). Carbon isotope composition (δ^13^C) of leaf dry matter and soluble sugars was calculated according to [46] as follows:

δ^13^C = R_s_/R_std_ − 1, (4)

where R_s_ and R_std_ are the isotope ratio (^13^C/^12^C) of samples and the international standard Vienna-Pee Dee Belemnite (VPDB), respectively. Carbon isotope discrimination of leaf dry matter and soluble sugars was calculated as:

Δ = (δ^13^C_a_ − δ^13^C_s_)/(1 + δ^13^C_s_), (5)

where δ^13^C_a_ is the carbon isotope composition of samples (leaf dry matter or soluble sugars) and δ^13^C_a_ is the carbon isotope composition of source air CO_2_, assumed to be about –9.5‰ on the basis of several measurements of air samples collected within the growth chamber [46].

### 2.9. Statistical Analysis

The statistical analysis was performed using GraphPad Prism, version 6.00 (GraphPad Software, La Jolla, CA, USA). One-way analysis of variance (ANOVA) followed by Fisher’s least significant difference (LSD) test was used to determine the significant difference between the means, assuming *p* ≤ 0.05 as the significance level.

## 3. Results

### 3.1. Hormonal Content in the Xylem Sap

**Figure 1:**
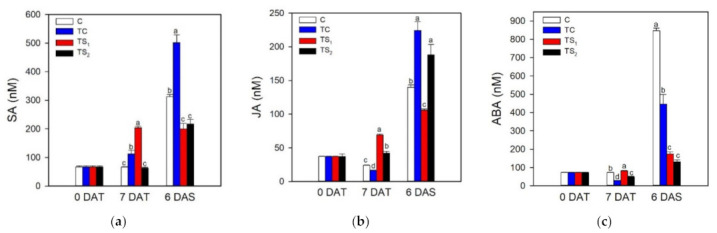
Hormonal content (nM) in the xylem sap of purple basil cv. Red Rubin at 0 and 7 days after bacterial treatment (0 DAT and 7 DAT, respectively) and at 6 days after water stress (6 DAS): (**a**) salicylic acid (SA); (**b**) jasmonic acid (JA); (**c**) abscisic acid (ABA). Values are means ± SE (*n* = 3). Different letters indicate significant differences among bacterial treatments and control (one-way ANOVA with Fisher’s LSD test, *p* ≤ 0.05).

The xylem exudates of purple basil plants in response to treatments with *A. baldaniorum* Sp245 were analyzed by GC/MS for the content of plant hormones (SA, JA, ABA) at 0 DAT, 7 DAT, and 6 DAS. As reported in Figure 1, the interaction between the PGPR and basil plants affected the amount of SA, JA, and ABA in the xylem sap, and bacterial treatments significantly differed both at 7 DAT and 6 DAS.

Considering the patterns of hormonal content in the xylem sap from 0 DAT to 7 DAT, data obtained by GC/MS showed that SA was induced by TC but especially by TS_1_: SA content peaked at approximately 200 nM in TS_1_ at 7 DAT. Following a similar pattern, xylem JA content increased in TS_1_ after inoculation reaching the value of about 70 nM at 7 DAT. This trend was not observed for ABA content, which was not induced from 0 DAT to 7 DAT both in bacterial treatments and control, rather showing a slight decrease in TC and TS_2_. During water stress from 7 DAT to 6 DAS, data for xylematic hormonal content indicated a marked induction of SA both in TC and C, reaching at 6 DAS values of about 310 and 500 nM, respectively. Lower increases of SA levels during water stress (200 nM) were observed in TS_1_ and TS_2_ than TC and C; moreover SA content in TS_1_ remained virtually unchanged at 6 DAS in comparison with its level before water stress. Xylem JA content increased both in bacterial treatments and control during water stress; at 6 DAS the higher JA levels were found in TC (more than 220 nM) and TS_2_. It is worth to note that JA in TS_1_ constantly increased for the whole duration of the trial from 0 DAT to 6 DAS. ABA was generally induced during water stress both in bacterial treatments and control, even though its increase was far more evident in TC and C. In particular, at 6 DAS xylem ABA content peaked in C (about 850 nM) and TC (about 450 nM), whereas a much lower ABA content was found in the xylem sap of TS_1_ and TS_2_ plants treated with filtered culture supernatants (about 170 and 130 nM, respectively). 

### 3.2. Leaf Water Potential 

**Figure 2:**
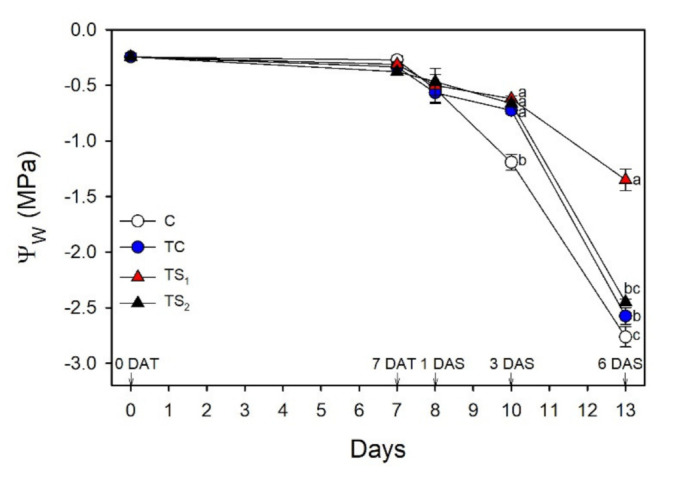
Leaf water potential (ψ_w_) in purple basil cv. Red Rubin at 0 and 7 days after bacterial treatment (0 DAT and 7 DAT, respectively) and at 1, 3 and 6 days after water stress (1 DAS, 3 DAS and 6 DAS, respectively). Values are means ± SE (*n* = 6). Different letters indicate significant differences among bacterial treatments and control (one-way ANOVA with Fisher’s LSD test, *p* ≤ 0.05).

Figure 2 reports leaf water potential (Ψ_w_) after inoculation and during water stress.

No significant differences of Ψ_w_ were observed from 0 DAT to 7 DAT, while after water removal Ψ_w_ progressively decreased in all bacterial treatments and control. At 1 DAS, a similar decrease in Ψ_w_ was observed in all treatments and control, while starting at 3 DAS a different behavior among bacterial treatments and control was evident. In particular, C showed a significantly lower Ψ_w_ value than the other bacterial treatments at 3 DAS. At 6 DAS, TS_1_ induced a significantly higher Ψ_w_ compared to the other bacterial treatments, while C showed the lowest value. 

### 3.3. Leaf Pigments (Chlorophylls, Total Carotenoids and Total Anthocyanins)

**Figure 3:**
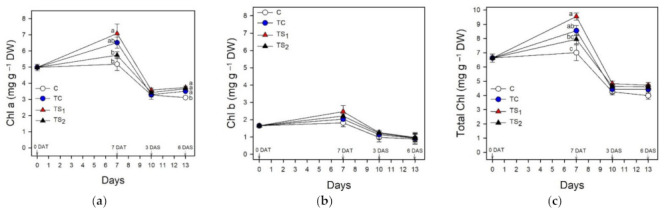
Leaf contents of chlorophylls in purple basil cv. Red Rubin at 0 and 7 days after bacterial treatment (0 DAT and 7 DAT, respectively) and at 3 and 6 days after water stress (3 DAS and 6 DAS, respectively): (**a**) chlorophyll a (Chl a); (**b**) chlorophyll b (Chl b); (**c**) total chlorophyll (total Chl). Values are means ± SE (*n* = 3). Different letters indicate significant differences among bacterial treatments and control (one-way ANOVA with Fisher’s LSD test, *p* ≤ 0.05).

The association of the PGPR with purple basil plants increased the levels of leaf pigments compared to controls and TS_1_ was the most effective bacterial treatment in this respect. Leaf chlorophylls of purple basil cv. Red Rubin treated with *A. baldaniorum* Sp245 are reported in Figure 3.

**Figure 4:**
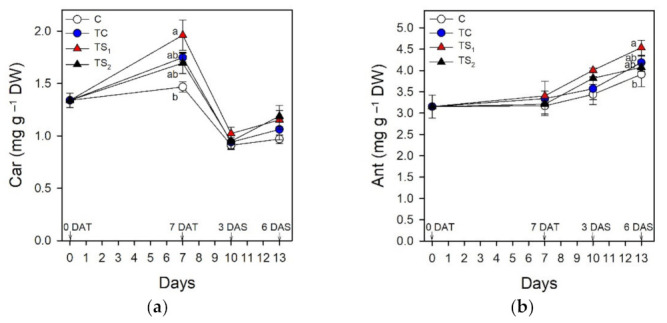
Leaf contents of total carotenoids and anthocyanins in purple basil cv. Red Rubin at 0 and 7 days after bacterial treatment (0 DAT and 7 DAT, respectively) and at 3 and 6 days after water stress (3 DAS and 6 DAS, respectively): (**a**) total carotenoids (Car); (**b**) total anthocyanins (Ant). Values are means ± SE (*n* = 3). Different letters indicate significant differences among bacterial treatments and control (one-way ANOVA with Fisher’s LSD test, *p* ≤ 0.05).

The highest Chl contents (a, b, total) were detected in TS_1_ at 7 DAT (7.08, 2.46, 9.54 mg g^−1^ DW, respectively). Drought stress was imposed at 7 DAT, completely removing water from the tanks. Chl decreased in each treatment during dehydration, dropping on average 49.3% in TS_1_ from 7 DAT to 3 DAS. Car (Figure 4a) were 1.33 mg g^−1^ DW at 0 DAT and peaked in TS_1_ at 7 DAT (1.96 mg g^−1^ DW), while during water stress Car decreased by an average of 47.3%. Ant (Figure 4b) were 3.15 mg g^−1^ DW before the bacterial treatment at 0 DAT and they incremented for the whole duration of the trial from 0 DAT to 6 DAS. TS_1_ induced the highest Ant content, which increased on average by 33.1% during water stress, reaching 4.53 mg g^−1^ DW at 6 DAS.

### 3.4. Photosynthetic Performance

**Figure 5:**
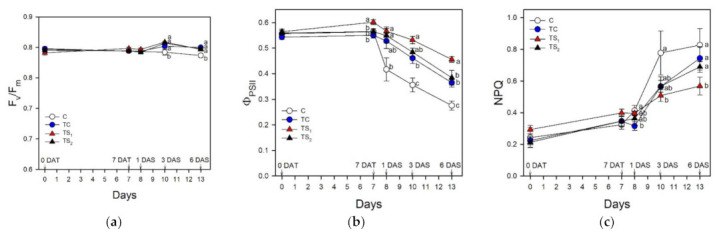
Chlorophyll fluorescence measurements in leaves of purple basil cv. Red Rubin at 0 and 7 days after bacterial treatment (0 DAT and 7 DAT, respectively) and at 1, 3 and 6 days after water stress (1 DAS, 3 DAS, and 6 DAS, respectively): (**a**) maximum PSII photochemical efficiency in the dark-adapted state (F_v_/F_m_); (**b**) actual photon yield of PSII photochemistry (Φ_PSII_); (**c**) non-photochemical quenching (NPQ). Values are means ± SE (*n* = 10). Different letters indicate significant differences among bacterial treatments and control (one-way ANOVA with Fisher’s LSD test, *p* ≤ 0.05).

The analysis of chlorophyll *a* fluorescence is reported in Figure 5. Fluorescence parameters F_v_/F_m_ and NPQ did not show any significant change at 7 DAT independently from bacterial treatments, while Φ_PSII_ was higher in TS_1_.

During water stress, F_v_/F_m_ was slightly lower in C compared to other bacterial treatments from 3 DAS. A decrease of Φ_PSII_ (associated with an increased NPQ) was observed in all plants during stress period, although with significant differences among bacterial treatments and control. In particular, starting from 1 DAS, Φ_PSII_ in C was lower compared to plants treated with viable cells (TC) and filtered supernatants (TS_1_, TS_2_). Subsequently, from 3 DAS to 6 DAS, TS_1_ and C showed the highest and the lowest Φ_PSII_ values respectively, while TC and TS_2_ displayed an intermediate value. NPQ concomitantly increased in all plants, although it was the lowest in TS_1_. 

**Figure 6:**
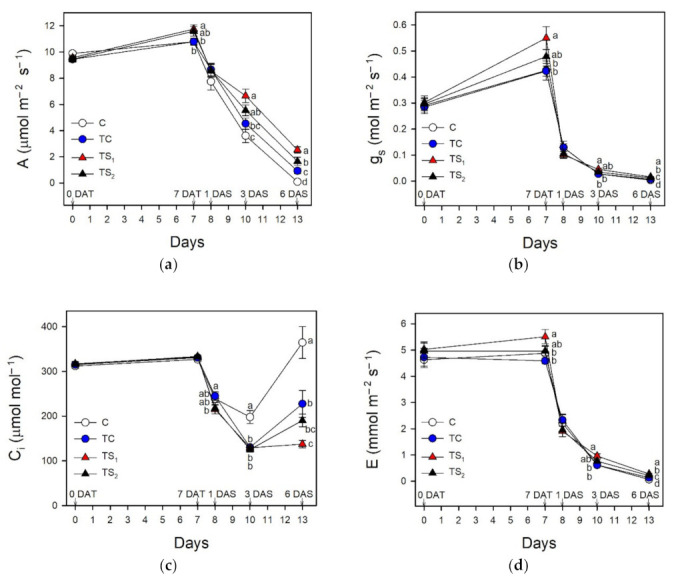
Gas exchange measurements in leaves of purple basil cv. Red Rubin at 0 and 7 days after bacterial treatment (0 DAT and 7 DAT, respectively) and at 1, 3, and 6 days after water stress (1 DAS, 3 DAS, and 6 DAS, respectively): (**a**) steady state photosynthetic CO_2_ assimilation rate (A); (**b**) stomatal conductance (g_s_); (**c**) intercellular CO_2_ concentration (Ci); (**d**) transpiration rate (E). Values are means ± SE (*n* = 10). Different letters indicate significant differences among bacterial treatments and control (one-way ANOVA with Fisher’s LSD test, *p* ≤ 0.05).

Figure 6 reports the gas exchange measurements in leaves during the bacterial treatment period and after water removal.

TS_1_ showed a significant increase of parameters A, g_s_, E compared to C and TC, while TS_2_ was not significantly different both from C and other bacterial treatments. Conversely, C_i_ did not show any significant difference during the bacterial treatment period. Water stress affected gas exchange parameters after 1 DAS, when all bacterial treatments and control exhibited a stress-induced reduction of A, g_s_, and E. Concomitantly, C_i_ significantly decreased in all bacterial treatments and control, ranging from the lowest in TS_1_ to the highest in C. A further stress-induced reduction of A, g_s_, and E was observed at 3 DAS and 6 DAS, with significant differences among bacterial treatments and control. In particular, at the end of the water stress period, A and g_s_ were the highest in TS_1_, followed in decreasing order by TS_2_, TC, and C. At 3 DAS, C_i_ in TS_1_, TS_2_, and TC was lower than C. At 6 DAS, C_i_ in C was higher than the other bacterial treatments; especially TS_1_, which showed the lowest value.

**Figure 7:**
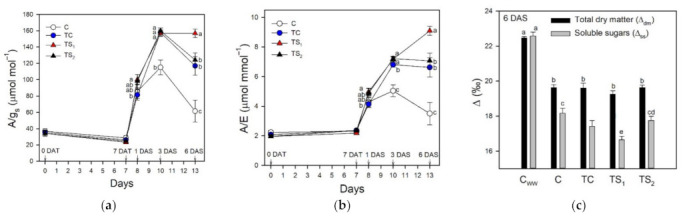
Water-use efficiency (WUE) and carbon isotope discrimination in leaves of purple basil cv. Red Rubin: (**a**) intrinsic WUE (A/g_s_); (**b**) instantaneous WUE (A/E); (**c**) carbon isotope discrimination (Δ) of leaf dry matter (Δ_dm_) and leaf soluble sugars (Δ_ss_). A/g_s_ and A/E were determined at 0 and 7 days after bacterial treatment (0 DAT and 7 DAT, respectively) and at 1, 3, and 6 days after water stress (1 DAS, 3 DAS, and 6 DAS, respectively); Δ_dm_ and Δ_ss_ were determined at 6 DAS and compared to well-watered plants (C_ww_). Values are means ± SE (*n* = 10 for A/g_s_ and A/E; *n* = 6 for Δ_dm_ and Δ_ss_). Different letters indicate significant differences among bacterial treatments and control (one-way ANOVA with Fisher’s LSD test, *p* ≤ 0.05).

The effects of treatments with *A. baldaniorum* Sp245 and water stress imposition on intrinsic and instantaneous WUE are shown in Figure 7a,b, respectively.

At 7 DAT, bacterial treatments did not affect both intrinsic and instantaneous WUE, while these parameters were significantly enhanced in treated plants after water removal. At 1 DAT all bacterial treatments and control increased their intrinsic and instantaneous WUE, especially in TS_1_. This was confirmed at 3 DAS, when both intrinsic and instantaneous WUE were significantly higher in TS_1_, TS_2_, and TC compared to C. At 6 DAS, the highest WUE values were found in TS_1_, followed in decreasing order by TS_2_, TC, and C.

### 3.5. Carbon Isotope Discrimination 

The effect of water stress on Δ_dm_ and Δ_ss_ is reported in Figure 7c. The Δ values decreased at 6 DAS in all bacterial treatments and control compared to the well-watered plants (C_ww_). While Δ_dm_ did not significantly differ among bacterial treatments and control, Δ_ss_ was differently affected depending on the bacterial treatment. In particular, the lowest Δ_ss_ was recorded in TS_1_, while C showed the highest value.

## 4. Discussion

### 4.1. Effects of Treatment with A. baldaniorum Sp245 on Hormonal Signaling in the Xylem Sap

Rhizosphere bacteria, such as *Azospirillum* spp., promote plant growth via several mechanisms including the production or degradation of the major groups of plant hormones that directly impact on plant growth and performance and reduce stress susceptibility [47]. Most of the evidence that PGPR produce or metabolize phytohormones in vitro has not always been translated into measurements of hormone concentrations in planta [48]. With the aim to link *A. baldaniorum* Sp245 treatments with the presence of some plant hormones that acts as root-to-shoot long-distance signals, we analyzed the presence of stress-related phytohormones (SA, JA, and ABA) in basil xylem exudates after the application of viable cells or filtered culture supernatants of *A. baldaniorum* Sp245 to the basil roots. Hormonal analysis of the xylem sap of basil plants grown under well-watered conditions and treated with viable cells or filtered culture supernatants (full dose) of *A. baldaniorum* Sp245 showed an increase of xylematic SA compared to untreated plants, especially in TS_1_, while after dehydration a sharp increase of SA was found in plants treated with viable cells. Recently SA was detected as a major molecule, among others plant hormones, in the supernatant of two *A. baldaniorum* strains [29]. The authors argued that the benefits of inoculation with *Azospirillum*, as well as leaf spraying of *Azospirillum* metabolites, on maize seeds or plants, were strongly correlated with the synthesis of phytohormones eliciting plant resistance to biotic and abiotic stresses. It is well known that the onset of systemic acquired resistance (SAR) is associated with increased levels of SA and is characterized by the coordinate activation of a specific set of PATHOGENESIS-RELATED (PR) genes, many of which encode PR proteins with antimicrobial activity [49]. Most of the studies on beneficial microbe-induced resistance point to a role for JA in the regulation of the induced immune response (ISR), but several examples of PGPR that trigger the SA-dependent SAR response have been also documented [47,49]. Incubation of basil plants with *Azospirillum* and application of SA decreased the drought effects and resulted in increasing of the growth [26]. Two studies [22,50] reported that exogenous application of SA enhanced drought stress tolerance in sweet basil plant. Our results highlight that basil plants treated with *A. baldaniorum* Sp245 (viable cells and filtered culture supernatants) increase the amount of SA in the xylem sap that transported to the aerial parts of the plants may affect plant performance under both well-watered and stress conditions. 

Xylem JA content was lightly affected only by filtered culture supernatants in basil plants grown under well-watered conditions; while after dehydration of the plants a marked increase of JA in response to the viable cells and TS_2_ was observed. JA was identified in the supernatant of two *A. baldaniorum* strains, but in relatively low amounts compared to other plant hormones [29]. ISR triggered by PGPR is often not associated with enhanced biosynthesis of JA but is mediated by altered JA sensitivity [49]. Several studies have also attributed an important role of PGPR in conferring to plants the ability to tolerate abiotic stress, by activating several physiological and biochemical mechanisms in plants, named induced systemic tolerance (IST) [51]. IST mechanisms include antioxidant defense, osmotic adjustment, production of phytohormones, and induction of heat-shock proteins [29]. Among plant hormones, JA is involved in the development of IST [52]. Several bacterial strains isolated from sunflower were able not only to produce JA, but also to increase content of this hormone when exposed to water stress [53]. Our results clear demonstrate that basil plants treated with filtered culture supernatants of *A. baldaniorum* Sp245 increase the amount of xylem JA translocated to the shoot, affecting plant growth under both well-watered and stress conditions. 

ABA level in xylem sap of basil plants grown under well-watered conditions and treated with viable cells or filtered culture supernatants of *A. baldaniorum* Sp245 remained substantially unchanged in comparison with untreated plants. After dehydration, a massive increase of ABA was found in untreated plants. ABA level also increased in basil plants treated with viable cells and filtered culture supernatants but in lesser amount. *A. baldaniorum* Sp245 produces ABA in vitro in a chemically defined medium, demonstrating that this bacteria can synthesize ABA as part of their normal metabolism [2]. Leaf ABA level has been investigated in maize [3] and in *A. thaliana* L. [8] inoculated with *A. baldaniorum* Sp245. These authors observed that the highest ABA level in leaves of both plants was induced by the combination of drought plus inoculation with *A. baldaniorum* Sp245, suggesting the presence of a synergism between the PGPR and water stress in increasing ABA levels. A marked accumulation of ABA was also observed in two varieties of green basil under salt stress [54]. In contrast with the results reported above by Cohen et al. [3,8], our data clearly shows an impaired increase of ABA level in xylem sap after dehydration of basil plants inoculated with viable cells and filtered culture supernatants of *Azospirillum*. Recently Belimov et al. [55] isolated and characterized rhizosphere bacteria capable of metabolizing ABA and decreasing ABA concentrations in planta even if the biochemical mechanisms have not been elucidated.

To the best of our knowledge, these represent the first results reporting the effects of root treatments with *A. baldaniorum* on the endogenous level of hormones in xylem sap and, taken together, suggest that the plant-rhizobacteria association significantly changes the level of the stress-related phytohormones SA, JA, and ABA, which could represent a priming mechanism able to affect the physiological responses and the tolerance to biotic and abiotic stress in purple basil.

### 4.2. Effect of Treatment with A. baldaniorum Sp245 on Leaf Pigments

Leaf chlorophylls, total carotenoids, and anthocyanins were determined during the trial to investigate the effects of bacterial treatments on photosynthetic and auxiliary photoprotective pigments in purple basil plants under well-watered and water stress conditions. Treatments with *A. baldaniorum* Sp245 increased leaf Chl in purple basil throughout the whole trial (Figure 3). The highest dose of filtered supernatants TS_1_ proved to be the most effective in this regard, even if significant differences among bacterial treatments and control resulted at 7 DAT for Chl a, total Chl, and Car and at 6 DAS for anthocyanins (Figure 3 and Figure 4). Total Chl content was maximum in TS_1_ at 7 DAT (9.54 mg g^−1^ DW). Chl (a, b, total) before bacterial treatments (4.98, 1.65, 6.63 mg g^−1^ DW, respectively) were consistent with data in literature [14,56,57,58,59,60]. Despite the research on the increase of photosynthetic and auxiliary photoprotective pigments induced by *A. baldaniorum* in other plants [8,61], studies on purple basil inoculated with *A. baldaniorum* are lacking. The only data showing a rise of Chl in basil treated with *A. baldaniorum* were obtained on green basil treated with an unspecified strain [62] and on an unstated basil variety inoculated with Sp245 [24]. Starting from 7 DAT, severe water stress conditions determined a major loss of Chl in each bacterial treatment and control, as previously found in basil [63,64,65,66]; the degradation of Chl during drought depends on the instability of protein complexes and on the increased activity of chlorophyllase and reactive oxygen species (ROS) [22,49]. Total Chl in TS_1_ decreased of 49.3% from 7 DAT to 3 DAS, larger than data by Damalas [22] with a milder stress. Even so, as reported for basil treated with different *Azospirillum* spp. [26,67], the loss of Chl during water stress was limited by *A. baldaniorum* Sp245 (Figure 3), since total Chl in TS_1_ was higher than controls both at 3 and 6 DAS (9.11% and 18.32%, respectively). Carotenoids (Figure 4) were lower than Chl, even if positively correlated (14). Car content before bacterial treatments (1.33 mg g^−1^ DW) was consistent with previous data [14,58,68]. Car levels were higher in treated plants but decreased during water stress, as also reported in [64] and [66]. The ratio Car/total Chl, a stress indicator [69], was in fact 0.20 at 7 DAT and 3 DAS, and 0.24 at 6 DAS. 

The purple color in basil morphotype *purpurascens* such as Red Rubin is due to the accumulation of coumaroyl and malonyl anthocyanins [14,57,58]. Total anthocyanins increased during water stress (Figure 4); their range (3.15–4.53 mg g^−1^ DW) was comparable to other purple basil varieties [70,71]. Anthocyanin content at 0 DAT was 3.41 mg g^−1^ DW; the fact that anthocyanin levels in basil markedly change depending on environment, extractions, plant maturity and material may explain the differences with other data [15,17,71]. Acting as ROS scavengers and osmoregulators, leaf anthocyanins are induced by abiotic stresses [19,72] and up-regulated in water-stressed plants [73,74]; anthocyanins also increase in purple basil under drought [21,63] and are positively correlated with drought tolerance in *Arabidopsis* [19]. *Azospirillum* spp. activate induced systemic tolerance to abiotic stresses and *A. baldaniorum* elicits drought tolerance [5,10,75]. Anthocyanins are induced by *A. baldaniorum* in bean and pepper [9], but researches on purple basil are still lacking. In our study, the maximum content of anthocyanins was in TS_1_ throughout the whole trial. During water stress, anthocyanins increased on average 33.1% in TS_1_ compared to 23.3% in C (Figure 4). Therefore *A. baldaniorum* Sp245 further enhanced leaf anthocyanins, which also increased in untreated plants during water stress. This is consistent with [8], in which the highest levels of anthocyanins in *Arabidopsis* were induced by the combination of drought plus inoculation with *A. baldaniorum* Sp245, suggesting a synergistic interaction between the PGPR and drought in increasing anthocyanins. Moreover, the increase of endogenous JA in xylem sap at 7 DAT in plants treated with filtered culture supernatants, especially in TS1, could promote the synthesis of anthocyanins during the following water stress period, in agreement with previous findings showing that the elicitation with exogenous JA treatments determined an increase of anthocyanins in purple basil leaves cv. Dark Opal [76]. Even if the complex interactions between this PGPR and basil need further research, our results suggest that treatments with *A. baldaniorum* Sp245 increased the leaf concentration of Chl and Car; while accumulation of anthocyanins was induced during water stress, concurring to plant-stress tolerance and also improving the nutraceutical value of leaves [77] in purple basil. 

### 4.3. Effects of Treatment with A. baldaniorum Sp245 on Photosynthesis and WUE

In order to understand the effects of bacterial treatments on the photosynthetic performance of purple basil under well-watered conditions and during water stress, gas exchange and chlorophyll fluorescence were monitored throughout the experimental trial. Our data show a beneficial effect of the highest dose of filtered culture supernatants of *A. baldaniorum* Sp245 (TS_1_) on photosynthetic CO_2_ uptake after 7 days of bacterial treatment (Figure 6). The improved photosynthetic activity was associated with significant higher values of Φ_PSII_ and g_s_, suggesting a stimulation effect of this bacterial treatment on both the energy conversion processes at PSII and stomatal aperture. This improved photochemical efficiency could be partly due to the increased content of photosynthetic pigments observed in TS_1_ plants. In support of this hypothesis, Kannan and Ponmurugan [78] reported an enhanced photosynthetic rate in rice varieties treated with *A. baldaniorum*, associated with a rise of total Chl, Car, soluble proteins and sugars. Chlorophylls, photoprotective photosynthetic pigments and growth rate also increased in wheat under optimal conditions inoculated with *A. baldaniorum* Cd. [61]. On the other hand, Ruiz-Sanchez et al. [79] showed that *Azospirillum* and arbuscular mycorrhizal (AM) colonization improved growth and physiological traits of rice under well-watered and drought conditions; in particular, co-inoculation with AM and *A. baldaniorum* caused an increased g_s_ of 35% and 80% under well-watered and drought conditions, respectively. 

Water deficit is one of the most important abiotic stresses affecting physiological and morphological traits in basil plants [63,80,81]. Given that basil has not been extensively studied yet, it is desirable to determine the effects of water availability on photosynthesis and productivity in economically relevant basil cultivars, especially in water-limited environments such as the Mediterranean area. Different basil cultivars showed a wide range of tolerance responses to water stress [82], suggesting that certain cultivars are more able to store water resources and thus more suitable to water-limited environments. The drastic reduction of leaf Ψ_w_ during water stress in purple basil cv. Red Rubin was associated with a decrease of g_s_ in attempts to reduce water loss due to leaf transpiration. As a consequence, photosynthesis decreased by stomatal closure in all bacterial treatments and control. It is worth noting that all plants safely dissipated the harmful excess energy at PSII as heat avoiding photoinhibition, as suggested by the concomitant increase of NPQ [83] and the maintenance of optimal F_v_/F_m_ values. During dehydration, Red Rubin plants treated with *A. baldaniorum*, especially with TS_1_, exhibited higher values of A and Φ_PSII_ compared to control plants. The positive effect of *A. baldaniorum* on the photosynthetic performance of basil plants during dehydration was reflected in a stable low C_i_ in treated plants after 6 DAS, while control plants showed a sharp increase of C_i_ indicating a drought-induced reduction of photosynthetic capacity. Moreover, plants treated with *A. baldaniorum* Sp245 showed an increased content of endogenous xylematic SA and JA that may alleviate oxidative stress in basil plants, as also reported by [29] on maize. Leaf treatments with SA reduced the detrimental effects of water deficit on photosynthetic pigments and growth parameters in basil [22,50,84]. Sorial et al. [85], investigating the responses of sweet basil to JA applications with different water supplies, highlighted its role in reducing the negative effects of water stress. Taken together, these results highlight a possible priming effect of *A. baldaniorum* on stress tolerance mechanisms in purple basil, affecting the endogenous hormonal signaling and improving both photosynthetic performance and leaf water status during dehydration, as also reported by [31] in wheat. The strong stomatal closure induced a reduction of transpired water to preserve the leaf turgor, although plants treated with *A. baldaniorum* maintained a slightly higher g_s_ compared to untreated plants, as previously observed [78]. The reduced g_s_ occurred concomitantly with a remarkable increase of xylem ABA, especially in C and TC plants (Figure 1). Conversely, TS_1_ and TS_2_ showed only a small increase of this hormone in the xylem sap, suggesting that ABA had only a marginal role in the stomatal regulation of stressed plants treated with filtered culture supernatants. Despite the stomatal closure and the reduced transpiration, all the stressed plants showed a progressive reduction of Ψ_w_, although TS_1_ better preserved leaf water status throughout the stress period (Figure 2). The improved water status of *A. baldaniorum*-treated plants was partly due to their capacity to increase the intrinsic and instantaneous WUE (Figure 7a,b) during the stress period. The increased WUE in basil plants may be due to greater decline of g_s_ (and transpiration) than reduction of photosynthesis during leaf dehydration. Even if all basil plants responded to dehydration by increasing WUE, this was particularly evident in TS_1_ plants. The increase of WUE is considered a crucial criteria to select appropriate cultivars and management systems (e.g., irrigation regime) in water limited environments [86], playing a key role to offset the impact of a changing climate [87]. In a study by Ekren et al. [81], purple basil was found to be sensitive to water stress and/or to the amount of irrigation water, although irrigation WUE did not significantly differ in response to water regime due to the concomitant decrease of both yield and the amount of water used for irrigation water. WUE in basil was also affected by the cultivar, with the highest WUE being observed in cvs. Mrs Burns and Cinnamon, and the lowest in cv. Red Rubin [80]. These results highlight that the selection of appropriate basil cultivars enhances WUE under water stress, saving water resources and allowing basil cultivation in areas with limited irrigation water. In the present work, the enhanced intrinsic and instantaneous WUE in cv. Red Rubin, especially in plants treated with *A. baldaniorum*, was supported by the decrease of Δ during water stress (Figure 7c). This parameter compared negatively to WUE in several species and it has been proposed as a criterion to select species and varieties more adapted to drought-prone environments [88]. In particular, Δ_dm_ gives a long-term integration of WUE, while Δ_ss_ estimates a short-term integration of WUE (few days), given that soluble sugars are mainly composed by recent photosynthates [89]. Hence, leaf soluble sugars were more representative of the drought stress period, and Δ_ss_ values were lower than Δ_dm_. Red Rubin plants inoculated with the highest dose of filtered culture supernatant of *A. baldaniorum* exhibited the lowest Δ_ss_ at the end of the trial, indicating that bacterial treatment TS_1_ was the most effective in inducing an increase of WUE during the last days of stress, according to the results obtained by gas exchanges. Considering both gas exchange and isotopic measurements, our results suggest that priming with *A. baldaniorum* Sp245 could be a promising approach for improving WUE in purple basil cv. Red Rubin and that the plant response is dependent on the bacterial treatment type and dose of filtered culture supernatant used, with the best results observed using the highest dose of filtered culture supernatant. The weaker effects observed in basil plants inoculated with viable cells with respect to those treated with filtered supernatants could indicate a poor root colonization by bacterial in our experimental conditions. Previous works showed that root colonization by *Bacillus subtilis* MBI 600 in cucumber plants was affected significantly by the growth substrate of the roots, with the lowest bacterial density observed in roots grown in grodan cubes placed in hydroponic floating systems with nutrient solution [90], which is a system similar to that used in the present study. Therefore, further research will be necessary to verify if this growing system affects the root colonization also of *A. baldaniorum* Sp245. However, these results suggest that applying supernatant instead of viable cells could represent a valuable tool for improving plant performance and stress tolerance when plants are grown in hydroponic system with grodan cubes as root substrate, which represent a widely used growing system in greenhouse conditions.

## 5. Conclusions

Our study indicates that the benefits of application of *Azospirillum* cells or their metabolites in purple basil can be attributed to the synthesis and transport of phytohormones that promote plant growth and confer tolerance to abiotic stress. Basil plant responses were dependent on the bacterial treatment type and the best results were observed using the highest dose of filtered culture supernatants of *A. baldaniorum* Sp245. This treatment increased leaf pigments (chlorophylls, total carotenoids, anthocyanins), enhancing both leaf water potential and photosynthetic performance during the water stress period. Finally, gas exchange and isotopic measurements clearly indicated an increase of intrinsic and instantaneous WUE during the water stress period in treated plants. Bacterial treatments based on *A. baldaniorum* Sp245 might represent a biological alternative to chemical inputs (fertilizers, pesticides, and plant growth regulators) with benefits also to the environment.

## Data Availability

All data and figures in the manuscript are original.
